# Oncology beyond borders: what global cancer care taught me

**DOI:** 10.1093/oncolo/oyag323

**Published:** 2026-08-13

**Authors:** Ivy Riano

**Affiliations:** Division of Medical Oncology, Dartmouth Cancer Center, Dartmouth Health, Geisel School of Medicine at Dartmouth College, Lebanon, NH 03756, United States

An observership in a Rwandan cancer clinic during my Medical Oncology fellowship transformed my understanding of oncology, showing me that exceptional cancer care depends as much on compassion as it does on access to treatment. Before Rwanda, I thought I understood adaptability in medicine. My training was in Colombia, Mexico, and the U.S. Onsite in Rwanda, I began to question what high-quality care means when resources are scarce; how clinicians function in multilingual practices; and what is the clinical North Star when “our” clinical algorithms are not appropriate?

I was eager to understand how Rwandan oncologists made decisions when evidence-based guidelines met real-world constraints.

My destination city of Kigali radiated resilience, energy and hope. At the *Rwanda Cancer Center*, I joined the outpatient clinic led by Dr. Fidel Rubagumya. In clinic, I was reminded how a patient’s expression, a shared smile, or gesture could be a powerful, if simple, common language. I was present, an attentive listener, and built human connections.

Like in my clinic at home, Rwandan patients’ decisions are shaped by disease, love, family, and values that do not always fit clinical algorithms. A young Rwandan mother with breast cancer arrived at clinic with a babe in arms. Her choice had been to delay treatment to breastfeed the child. I empathized with her situation and connected as I am also a mother with an infant. Her instinct to protect the child transcended a clinical flow chart.

The Rwandan proverb *When you are well, you belong to yourself. But when you are sick, you belong to your family* came alive in a family meeting for a patient with metastatic gastric cancer deteriorating dramatically. Family joined the discussion about prognosis and goals of care. They did not hold back. They were decision-makers and guardians of her dignity. I appreciated the family’s role in the experience of illness.

I experienced use of the “Safari Concept,” developed by Dr. Christian Ntizimira. This culturally grounded framework for end-of-life care uses animal archetypes to describe family roles and patterns of illness, suffering, and loss. It acknowledges that sometimes our role is not to cure, but to bring compassion and presence to families in difficult moments.

I witnessed collaboration among teams of surgeons, oncologists, and psychologists who functioned in single visit oncology. With everyone in the room, their multidisciplinary practice evaluated a patient using a holistic and practical approach. With limited resources, every decision carry weight. My appreciation for collaboration among physicians, nurses, and other healthcare professionals deepened.

Scarcity of drug was a common theme. Often medications like bevacizumab or trastuzumab were too expensive or delayed awaiting approvals. I saw oncologists advocate for patients waiting anxiously for treatment.

I realized that guidelines are a framework. Reality requires adjustments. Rwandan oncologists adapt based on availability and feasibility. Treatment for a 28-year-old woman with a diffuse large B-cell lymphoma should have been straightforward with R-CHOP (rituximab, cyclophosphamide, doxorubicin, vincristine, prednisone), but it required all components be in clinic, at the same time. A tall order. I observed clinicians flex to meet chemotherapy goals with sharing, trading, rethinking, and sometimes failing.

In imaging, computed tomography and PET-CT scans were limited and prioritized. Physicians weighed whether a test would meaningfully alter management. I saw the same scarcity in Colombia. It reminded me, in low-resource countries, evidence-based medicine is often trumped by resource-based medicine.

Now back at work, I find Rwandan lessons learned shape my attitudes and practice. My job is not just knowing the right answer, it’s determining what can realistically be accomplished and delivering care with intention and clarity. I pause before ordering tests or treatments, asking whether they will change a patient’s care and whether the anticipated changes are worth the burden.

Rwanda transformed how I view oncology care. I was inspired by the resilience, teamwork, and dedication of Rwandan colleagues. They showed me that quality cancer care should never depend on solely geography or technology.

Reflecting on this experience, I remain deeply grateful to the patients, families and colleagues who welcomed me into their lives. Their lessons continue to guide my practice and my understanding of what it means to care for people with cancer. *Murakoze cyane*, dear Rwandans—thank you for the enduring lessons you shared. They will stay with me throughout my career and my life.

